# Air–liquid intestinal cell culture allows *in situ* rheological characterization of intestinal mucus

**DOI:** 10.1063/5.0187974

**Published:** 2024-05-07

**Authors:** Pamela C. Cai, Margaret Braunreuther, Audrey Shih, Andrew J. Spakowitz, Gerald G. Fuller, Sarah C. Heilshorn

**Affiliations:** 1 Department of Chemical Engineering, Stanford University, Stanford, California 94305, USA; 2 Department of Materials Science and Engineering, Stanford University, Stanford, California 94305, USA

## Abstract

Intestinal health heavily depends on establishing a mucus layer within the gut with physical properties that strike a balance between being sufficiently elastic to keep out harmful pathogens yet viscous enough to flow and turnover the contents being digested. Studies investigating dysfunction of the mucus layer in the intestines are largely confined to animal models, which require invasive procedures to collect the mucus fluid. In this work, we develop a nondestructive method to study intestinal mucus. We use an air–liquid interface culture of primary human intestinal epithelial cells that exposes their apical surface to allow *in situ* analysis of the mucus layer. Mucus collection is not only invasive but also disrupts the mucus microstructure, which plays a crucial role in the interaction between mucus and the gut microbiome. Therefore, we leverage a noninvasive rheology technique that probes the mechanical properties of the mucus without removal from the culture. Finally, to demonstrate biomedical uses for this cell culture system, we characterize the biochemical and biophysical properties of intestinal mucus due to addition of the cytokine IL-13 to recapitulate the gut environment of *Nippostrongylus brasiliensis* infection.

## INTRODUCTION

Mucus covers every wet epithelial surface of the body, from the eyes to the lungs to the gastrointestinal tract.^1–5^ As the first barrier to our inner organs, mucus must be sufficiently viscous to protect the epithelium against shear forces but also elastic enough to be a selective physicochemical barrier against foreign or harmful molecules and pathogens. Mucus accomplishes these goals by forming a polymer network composed mainly of glycoprotein mucins, which can form reversible physical bonds either through hydrophobic interactions, chain entanglements, or electrostatic attraction.^6–8^ This network dictates the microstructure of mucus, which is important to nutrient diffusion and the dynamics of the gut microbiome.^2,9–12^ Furthermore, the chain associations are sensitive to pH, where low pH causes mucus to become an elastic gel and neutral pH causes mucus to flow like a viscous liquid.^8,13–15^ Any dysfunction in the physical properties of mucus can lead to disease and infection, such as mucus becoming less elastic at low pH and allowing pathogen diffusion to the epithelium to cause infection.^10,16^

Many studies directed at intestinal mucus properties involve animal models, which are expensive and require invasive procedures to obtain the mucus.^17,18^ These procedures involve scraping mucus from the extracted intestine, which disrupts the microstructure of the mucus gel and alters its physical properties. To circumvent these issues, others have used native intestinal mucins purified from pig stomachs, which appear to exhibit the expected viscoelastic behavior unlike commercially available purified porcine gastric mucin.^19,20^ However, we hypothesized that the lack of attachment to the epithelium would significantly alter the dynamics of the mucin gel. Furthermore, the absence of cells does not allow for biological perturbations to the system.

An *in vitro* cell culture system with an accessible intestinal mucus layer is a way to combat the shortcomings of other methods to study intestinal mucus. Although successful growth of a cell monolayer and mucus layer using intestinal cells has been demonstrated using the “organ-on-a-chip” technology, this technique lacks direct access to the mucus layer for mechanical measurements of the fluid.^21^ Another cell-culture method—the air–liquid interface culture—has been successfully implemented for primary human lung and gastric stem cells, as well as colon cell lines, to form robust cell and mucus layers.^22–26^ Here, the air side of the culture develops apical polarity and provides access to the mucus layer for measurements of physical properties like creep compliance and mucociliary clearance velocity, which are directly related to the movement of nutrients and gut microbiota found in the intestinal mucus layer. It is therefore advantageous to leverage the air–liquid interface culture for direct access to the mucus layer, and combining this with intestinal cells and novel rheological techniques can reveal insights into the physics of the intestinal mucus layer.

In this work, we developed an air–liquid interface culture system for primary human intestinal stem cells that allows rheological characterization of intestinal mucus without perturbation to the mucus microstructure. This system can also be leveraged to study the effect of diseases on intestinal mucus. Especially prevalent in countries with poor hygiene, gastrointestinal infection with the helminth *Nippostrongylus brasiliensis* (*N. brasiliensis*) poses a significant health burden, and treatment strategies require better understanding of the host immune response.^27^ However, the physical effects of the immune response, particularly on intestinal mucus, and the associated impact on infection progression are not well understood. We investigated the impact of *N. brasiliensis* infections on intestinal mucus through the addition of cytokine IL-13, which prior work shows introduces perturbations akin to *N. brasiliensis* infections.^28^ We hypothesized that, similar to lung infections, helminth infections would lead to physical changes in mucus properties.^29^ To probe these changes, we used a nondestructive rheological method that leaves the native microstructure intact during measurement. This work is a proof of concept for a platform that future investigations can leverage to study intestinal disorders affecting mucus biophysics.

## RESULTS

### Development of intestinal mucus layer cultures

**FIG. 1.**
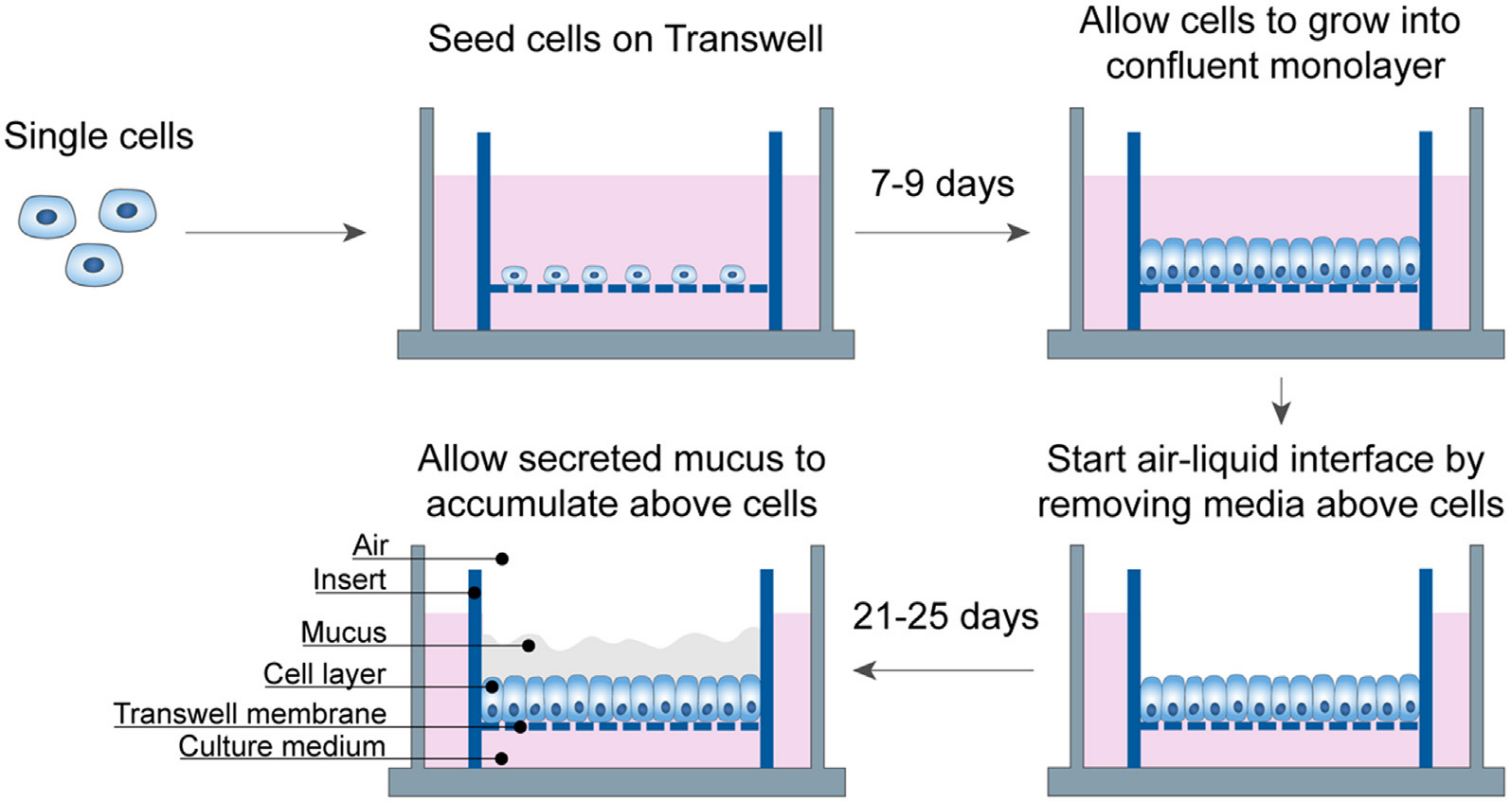
Protocol to develop air–liquid interface culture of primary intestinal cells with a robust mucus layer. Single cells from primary donor duodenum tissue are seeded onto a Transwell insert and cultured for 7–9 days while submerged in differentiation medium. Once cells have grown into a confluent monolayer, the differentiation medium is removed above the cells and continued to be replenished below the Transwell. After 21–25 days of air–liquid interface culture, a robust mucus layer will be established above the cell layer.

The mucus layer plays a crucial role in the intestines as it is the home and nutrient source for microbes in our gut. Since mucus lies on the epithelial surface of the intestines, studying the mucus microenvironment *in vitro* requires polarization of intestinal cells to trigger mucus secretion into a robust layer, thereby mimicking the biophysical environment experienced by microbes in the gut. Culturing an intestinal mucus layer began with isolating intestinal stem cells from healthy patient intestinal duodenum. Based on previous work culturing primary human gastric cells at an air–liquid interface, we hypothesized that using the same culture method for intestinal stem cells would lead to the same polarization of the epithelium and growth of a mucus layer.^24^ The intestinal stem cells were first cultured on Matrigel-coated Transwell inserts while submerged in differentiation medium. Next, the differentiation medium was removed from above the cells inside the Transwell after a confluent layer formed (Fig. 1), beginning the air–liquid interface culture period. The mucus layer accumulated above the cell layer over the next 3 weeks into a robust mucus layer.

**FIG. 2.**
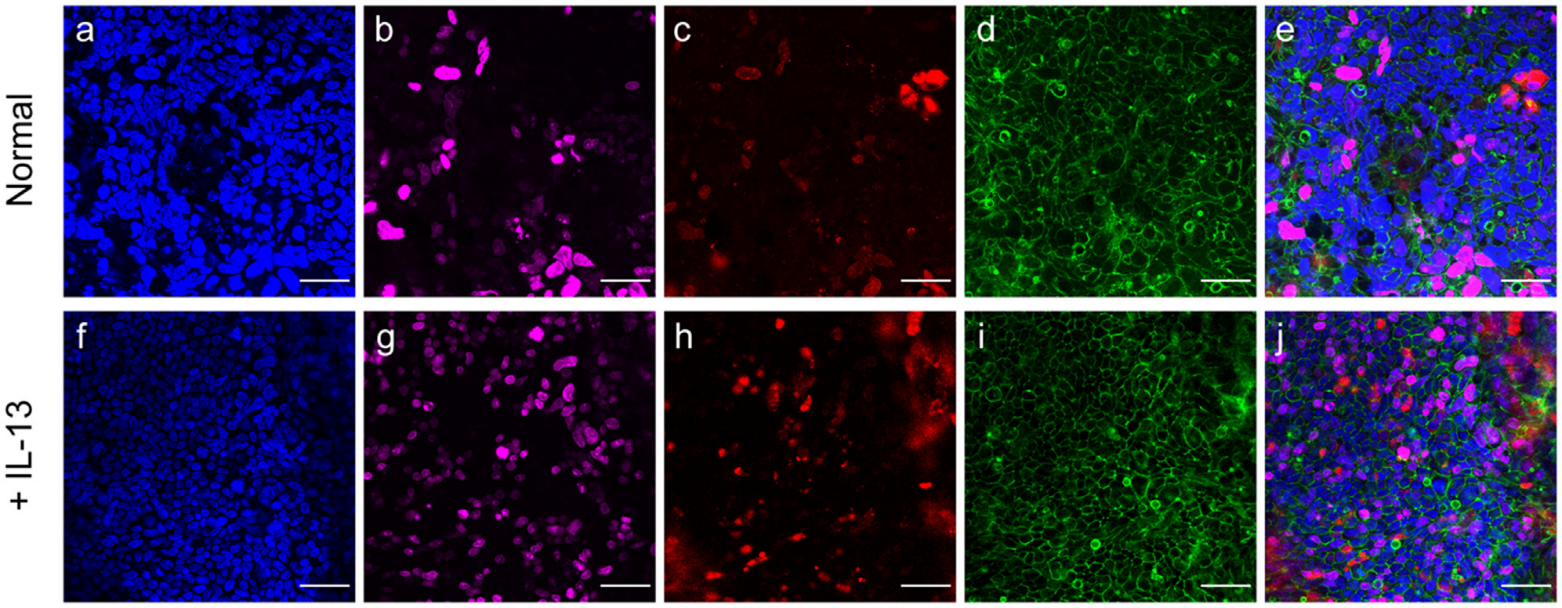
Immunofluorescent staining of intestinal air–liquid interface cultures. Intestinal cell monolayers after 21–25 days of air–liquid interface culture were fluorescently stained for (a) DAPI (nuclei), (b) Ki-67 (proliferation), (c) MUC2 (mucin), and (d) phalloidin (cell boundary). The merged image of all the stains is shown in (e). Staining for cultures also given inflammatory cytokine IL-13 are shown in (f)–(j). Scale bar: 50 *μ*m.

Immunofluorescent staining of fixed and permeabilized samples confirmed the formation of a confluent monolayer of cells on the Transwell membrane [Figs. 2(a) and 2(e), supplementary material, Movie 1]. Several cells were positive for proliferation marker Ki-67 [Fig. 2(b)], demonstrating that this culture platform supports continued cell growth. Although there are 21 mucin-type glycoproteins that belong to the *MUC* gene family in humans, the main mucin secreted in the intestines is *MUC2* (mucin-2), which in the small intestine is typically anchored to goblet cells right after secretion.^1,11,30,31^ Immunostaining revealed *MUC2* in localized regions throughout the culture [Fig. 2(c)], confirming goblet cell differentiation as expected due to the addition of N-[(3,5-difluorophenyl)acetyl]-L-alanyl-2-phenyl]glycine 1,1-dimethylethyl ester (DAPT) in the medium. Polarization of the cell layer is also apparent in a sideview image of the cultures, showing that the *MUC2* staining is mainly present above the cell layer (supplementary material, Fig. 1).

### Model of *N. brasiliensis* worm infection in intestines

Gastrointestinal helminth infections are a global health problem and remain prevalent in countries with poor hygiene. Infection with the helminth *N. brasiliensis* poses a significant health burden particularly to children because reinfections are very common and can cause morbidity and mental retardation.^27^ Previous work has shown that RNA-seq on duodenal epithelium from mice infected with the worm *N. brasiliensis* exhibit similar genetic signatures to treatment of intestinal organoids with cytokine interleukin IL-13.^28^ As we use cells from the donor duodenum in our cell culture model, administration of IL-13 to our cultures may recapitulate the *N. brasiliensis* infection conditions. In other organs, such as the lungs, infections can alter the physical properties of mucus, such as becoming more elastic and possessing smaller pore sizes that limit nutrient diffusion.^32,33^ Specifically, airway epithelial cells treated with IL-13 exhibited slower mucociliary beat frequency, suggesting that mucus fluid becomes thicker and more elastic.^29,30^ We hypothesized that an IL-13-induced infection model of the intestines would reveal similar changes in mucus rheology. In other words, treatment with the inflammatory cytokine IL-13 would lead to a more elastic intestinal mucus.

Mucins are mainly secreted by goblet cells. These cells are differentiated from stem cells through a series of biochemical signals along the Notch and Wnt pathways. Specifically, inhibiting Notch and Wnt signals can lead to goblet cell differentiation. Previous work showed that IL-33, a cytokine upregulated in inflammatory bowel disease and helminth infections like *N. brasiliensis*, induces intestinal goblet cells and *Muc2* expression in mice through IL-13 production by innate lymphoid cells.^34^ Thus, by adding IL-13 at concentrations found in mice infected with *N. brasiliensis*, we hypothesized that we could emulate the effect of innate lymphoid cells during infection and a similar upregulation of goblet cell genes and mucin genes would occur.^27,35^

**FIG. 3.**
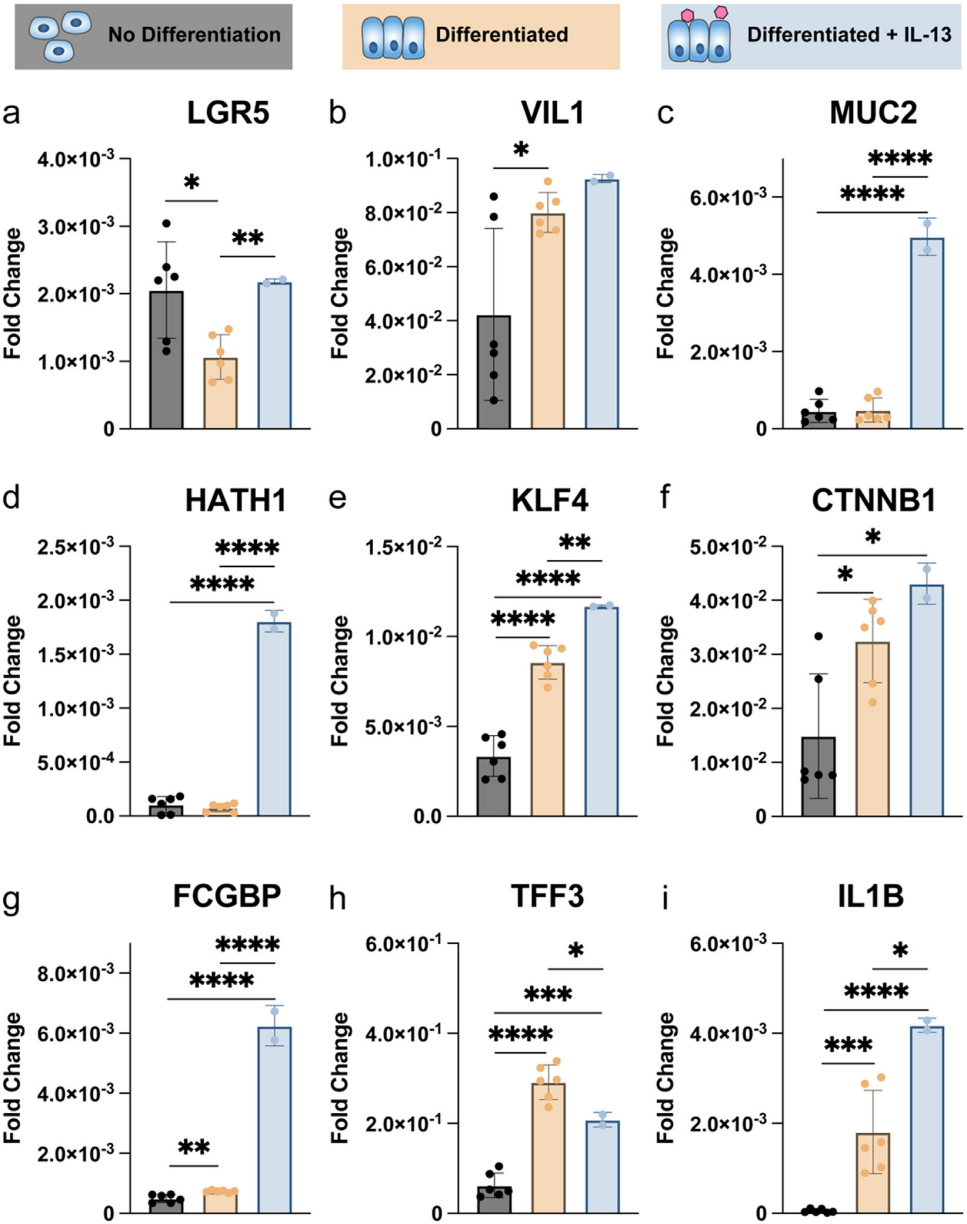
RNA analysis shows that the addition of IL-13 upregulates mucin-related genes. Quantitative PCR on cells that were not given differentiation medium (black, n = 6), cultured with differentiation medium (yellow, n = 6), or cultured with differentiation medium and treated with IL-13 (blue, n = 2) is shown for several clusters of genes: (a)–(c) shows the genes associated with intestinal cell type, (d)–(f) shows genes associated with goblet cell differentiation, and (g)–(i) shows genes related to mucin production. Paired samples t-test. ^*^p < 0.05, ^**^p < 0.01, ^***^p < 0.001, and ^****^p < 0.0001.

We compared the RNA-level differences between non-differentiated intestinal stem cells, differentiated intestinal cells cultured at an air–liquid interface, and IL-13 treated differentiated cells cultured at an air–liquid interface. As expected, we found that non-differentiated cells had higher *LGR5* (leucine-rich repeat-containing G-protein coupled receptor 5) expression, which is indicative of more stem-like cells, than differentiated cells [Fig. 3(a)].^36^ On the other hand, LGR5 expression appeared to be at similar levels between the non-differentiated and IL-13 treated differentiated cells, though the non-differentiated cells exhibited greater variance in LGR5 expression. For differentiated cells, both untreated and IL-13 treated cultures expressed higher levels of *VIL1* (villin-1), a marker for enterocytes, compared to non-differentiated cells [Fig. 3(b)]. *MUC2* expression is used as a marker of goblet cells, which was significantly higher in the IL-13 treated cells than the other two conditions [Fig. 3(c)].

If we look more closely at genes associated with goblet cell differentiation, we see similarly significant trends as *MUC2* expression. *HATH1* (atonal BHLH transcription factor 1) is expressed upstream of *SPDEF* (SAM pointed domain containing ETS transcription factor), which promotes the differentiation of secretory progenitor cells into goblet cells. *KLF4* (KLF transcription factor 4) also promotes the proliferation of mature goblet cells, as mice null for this gene displayed a 90% reduction of mature goblet cells in the colon.^37,38^ It must be noted that in this same study, higher up in the small intestine, goblet cell differentiation was minimally affected by knockdown of *KLF4*. Both *HATH1* and *KLF4* are most highly expressed in the IL-13 treated cultures [Figs. 3(d) and 3(e)]. *KLF4* expression in the untreated differentiated cultures is also significantly higher than the non-differentiated condition. In the intestines, canonical Wnt signaling has been linked to a reduction in goblet, enteroendocrine, and Paneth cells.^37^ However, *SPDEF* was previously identified as a downstream target of Wnt signaling, and we found that *CTNNB1* (catenin beta-1) expression, which is upregulated with Wnt signaling, increased with goblet cell differentiation [Fig. 3(f)].^39,40^ While genes associated with goblet cell proliferation were more highly expressed in the differentiated and IL-13 treated cultures, the role of Wnt signaling on goblet cell differentiation was less definitive.

Finally, we examined the transcription of several mucin-related genes in the IL-13 treated cultures compared to the other two conditions [Figs. 3(g) and 3(i)]. *FCGBP* (Fc Gamma Binding Protein) as well as TFF3 (trefoil factor 3) are secreted by intestinal goblet cells and cross-link mucins to form a network that acts as an effective physicochemical barrier.^41^ Interestingly, we see a higher expression of *TFF3* in the untreated cells compared to the IL-13 treated cells [Fig. 3(h)], but higher expression of *FCGBP* in the IL-13 treated cells compared to the untreated [Fig. 3(g)]. *IL1B* (interleukin-1 beta) indicates an inflammatory response, and high expression after an infection-simulating treatment is expected. We observed significant upregulation of *IL1B* in the IL-13 treated cultures compared to the other two conditions [Fig. 3(i)]. Overall, mucin-related genes were more highly expressed in the differentiated conditions, and we observed elevated levels of inflammation with IL-13 treatment.

Visually, we detected a slight increase in the amount of *MUC2* and Ki-67 staining in cultures treated with IL-13 [Figs. 2(g) and 2(h), supplementary material, Movie 2]. Otherwise, the immunofluorescent staining shows very similar confluent monolayers between the treated and untreated cultures despite RNA expression data suggesting upregulation of mucin-related genes [Figs. 2(f), 2(i), and 2(j)]. The staining process can wash away loose mucins, complicating the use of staining as a method of quantifying mucin production and its physical impact. Thus, to better assess the physical impact of any changes in mucin production, our next step was to characterize the physical properties of the mucus layer in a nondestructive manner.

### *In situ* rheological characterization of intestinal mucus

**FIG. 4.**
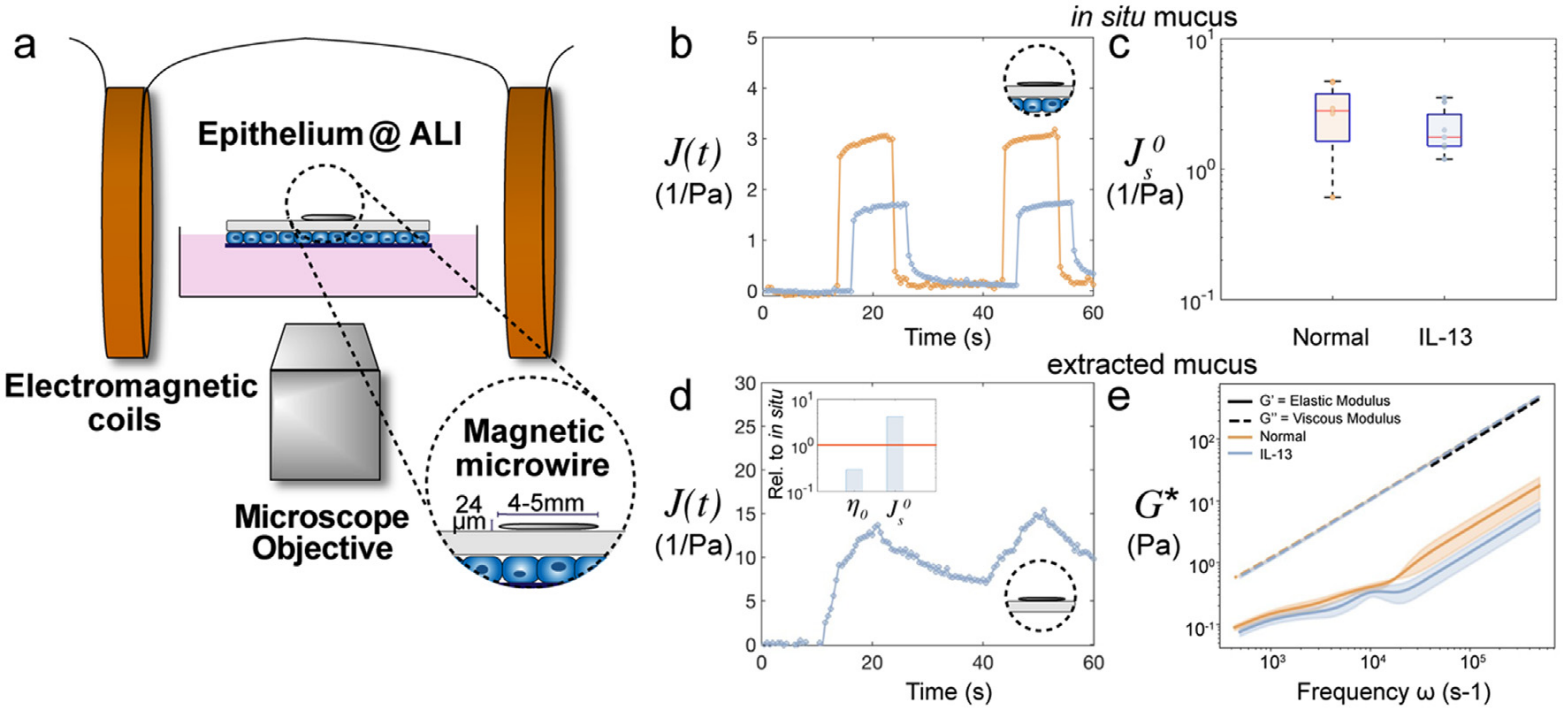
Rheological characterization of secreted mucus in intestinal air–liquid interface cultures. *In situ* rheological characterization of the mucus layer above the intestinal cell monolayer using a microwire is shown schematically in (a). Step creep compliance measurements over time on normal (yellow) and IL-13 (blue) treated intestinal cell mucus layers are shown in (b). The steady state compliance is shown in (c) for normal intestinal cell cultures and those treated with IL-13 (no statistical significance, n = 4); (d) is a step creep compliance measurement on extracted mucus fluid on a plastic surface instead of cells. The inset shows the relative zero-shear viscosity and steady state compliance for the mucus from IL-13 treated cultures on a plastic surface normalized to the measurements performed *in situ.* Finally, the mucus on normal intestinal cell cultures and those treated with IL-13 were extracted and rheologically characterized using dynamic light scattering microrheology. The resulting complex moduli are shown in (e). The black dotted line corresponds to the viscous modulus of pure water. We note that the viscous moduli (denoted by the yellow and blue dashed curves) for both normal and IL-13 are nearly identical and are very comparable to that of water.

Secretory mucins, particularly MUC2, are expelled from goblet cells as globules that expand and cross-link with other expanded globules to form large networks and give structure to the mucus layer.^31^ It is critical to study the physical properties of mucus without disrupting this network structure to truly grasp how these networks physically contribute to many roles of mucus within the human body, including being an antimicrobial barrier, and how mucus is impacted during disease. Thus, we leveraged a rheology technique that uses a magnetic field gradient to control the movement of a magnetic microwire probe, which sits at the surface of the mucus layer without destroying the fluid structure [Fig. 4(a)]. This device was developed to make nondestructive rheological measurements of biomaterials, as demonstrated on gelling and degrading hyaluronic acid hydrogels.^42^ Recently, this device was implemented to characterize the mucus on live human airway epithelial cell cultures *in situ* for the first time and quantify the change in mucus rheology with IL-13-induced inflammation associated with asthma.^43^ In that work, we demonstrated this technique as a powerful method to quantify the impact of disease on mucus material properties and elucidate the relationship between epithelial cell physiology and the secreted mucus rheology. The methods developed in that work were adapted here for *in situ* rheology on air–liquid interface cultures of intestinal epithelial cells.

Specifically, the entire Transwell containing the air–liquid interface culture of intestinal epithelial cells was placed between two electromagnetic coils, and a magnetic microwire probe was placed at the mucus and air interface. The device was mounted on an inverted microscope, allowing live imaging of the culture and microwire motion during the experiment. The coils generated a magnetic field gradient whose strength controlled the amount of force exerted on the microwire in the axial direction [Fig. 4(a)]. The translational displacement of the microwire on the surface was captured using a video camera (supplementary material, Movie 3). Finally, the magnitude of the microwire displacement relative to the applied step force was used to determine the creep compliance of the material, a measure of viscoelasticity.^42^ For example, a perfectly elastic solid would be displaced instantaneously when the force is turned on and returned to the original position instantaneously when the force is removed. On the other hand, a perfectly viscous liquid would move gradually, or flow, at a constant speed in the direction of the force and not display any recoil after the force is removed. Viscoelastic materials would exhibit a combination of the two extremes where both elastic deformation and viscous flow would each be present at different time scales. A viscoelastic solid would exhibit little to no long-time flow and a complete recovery, whereas a viscoelastic liquid would exhibit a stead-state behavior flow and a partial recovery due to energy partially being stored elastically, with the remaining portion dissipated via viscous flow. Techniques similar to the one used here have been developed for the measurement of interfacial shear rheology.^44–46^ Unlike prior similar techniques, our technique assumes that the microwire diameter (24 *μ*m) is large enough compared to the thickness of the mucus layer (∼30 *μ*m) that measurements characterize the bulk material behavior rather than interfacial properties.

Creep compliance measurements were performed on the mucus layer of normal and IL-13 treated air–liquid interface intestinal cell cultures. These measurements were performed at intervals of 10 s, followed by a relaxation period of 20 s, twice over the course of 1 min. Thus, the compliance data show a jump from 0 to a higher value during intervals of 10 s when the force is turned on [Fig. 4(b)]. The elastic deformation at the onset of applied force and the complete recovery after the removal of the stress indicate that the mucus layers of both the IL-13 treated cultures and the untreated cultures are elastic solids. The mucus layer of the IL-13 treated cultures appeared to have a lower compliance than the mucus of untreated cultures [Fig. 4(b), blue for IL-13 and yellow for untreated], indicating a stiffer material. Using the time-dependent compliance data, we derived a steady-state compliance and a zero-shear viscosity using the viscoelastic Burgers model.^42^ It appears that the introduction of IL-13 results in a small reduction in compliance, although the difference is not quite statistically significant [Fig. 4(c)]. There was no change in the measured zero-shear viscosity between the two conditions (supplementary material, Fig. 2).

We hypothesized that the presence of the cells would alter the viscoelastic properties of mucus because the mucins may be tethered to the cells. To test this idea, we performed creep compliance measurements of the mucus after extracting it from the cells and pipetting onto on a plastic surface [Fig. 4(d)]. It is evident from the compliance data that the extracted mucus is a viscoelastic liquid, as the material exhibited steady-state behavior of flow and only a partial recovery when the force was removed. Additionally, in a further contrast to the *in situ* measurements, the extracted mucus compliance was much greater, increased by a factor of 4, indicating a softer material. It is reasonable to suspect the removal of the mucus from the cells and transport onto the plastic dish could permanently alter mucus properties, as has been found to occur in biofilms, which exhibit reduced viscoelasticity when gently scraped onto a rheometer.^47^ Furthermore, the process of pipetting the mucus layer off the culture surface could leave behind the most adherent and solid-like mucus.^48^ Finally, we also characterized the extracted mucus properties using dynamic light scattering microrheology.^49^ We again saw more liquid-like behavior compared to the viscoelastic solid properties measured *in situ*. We found that there was very little difference between the extracted mucus and water [Fig. 4(e), black dotted line], which is not entirely surprising given that the solid content percentage was around 1.3% (supplementary material, Fig. 3). Airway mucus has been measured to be 3% solids, which could be greater than our measured value due to the additional DNA presence.^32^ These results demonstrate the need to measure mucus in contact with the epithelium and with limited disturbance since extracted mucus does not appear to exhibit the viscoelasticity it possesses *in vivo*.^17^

## DISCUSSION

This work demonstrates the first air–liquid interface culture of human primary intestinal cells into a confluent monolayer with a robust mucus layer that can be rheologically probed without disturbing the fluid structure. The *in vitro* nature of the model allows for perturbations to the system to model diseased conditions, which we demonstrated by adding IL-13 to the media to mimic *N. brasiliensis* infections. To our knowledge, there have never been attempts to probe the rheological changes in intestinal mucus arising from helminth worm infections. Our use of a magnetic microrheometer to directly measure the compliance of intestinal mucus after IL-13 treatment is the first approach to quantify the biophysical effects of helminth infections. This technique opens the possibility of exploring hypotheses put forward by previous studies of helminth infections. In particular, movement of intestinal mucus through the intestinal tube is the typical method for expelling foreign objects, but the mechanism by which helminths are expelled and how rheological changes in mucus can impact this process remain unknown. We also show that typical procedures of mucus extraction from animal or cell models for rheological studies is insufficient and inaccurate when such collection procedures permanently alter the underlying material microstructure and could leave behind the most adherent, solid-like mucus [Figs. 4(b) and 4(d)].

Although previous work showed that adding IL-13 to intestinal organoid cultures from human duodenal tissues exhibited similar gene expression to mice infected with *N. brasiliensis*, we were unable to detect major differences in the rheological properties of mucus from treated and untreated cultures. Our gene expression data did suggest an increase in goblet cell differentiation in the presence of IL-13. However, there was only a slight increase in MUC2 staining. This translated to the material properties of the mucus as there was also no significant difference between the steady-state compliance of the treated and untreated cultures. We thus conclude that our hypothesis that mucus production would increase due to IL-13 stimulation alone was incorrect. This result could be due to IL-13 not being enough by itself to trigger this mucin production response, which is possible as there has been work showing that IL-4 and IL-13 together are associated with production of intestinal mucus in mice.^35^ Another likely scenario is that IL-13 is typically secreted in the body by innate immune cells, such as eosinophils in the lungs, so perhaps IL-13 secretion is not the only contribution of these immune cells, which are lacking in our system, to mucin production.^27,50^ A next step is to co-culture intestinal stem cells with T-cells that would be triggered to secrete IL-4/IL-13 and observe whether mucin production increases. In mice, the role of innate IL-4/IL-13 has been implicated in goblet cell hyperplasia and worm expulsion, but the biophysics of how the expulsion occurs and whether it is due to fluid property changes in intestinal mucus needs to be further investigated. A co-culture system that could model the innate immune response and observed goblet cell differentiation seen *in vivo* could reveal mucus rheological changes that aid worm expulsion.

Overall, this cell culture system is an ideal platform for biophysical studies of the intestinal mucus layer. We have demonstrated just one example of how the platform can be leveraged for disease modeling. Given the co-culture capabilities and robust mucus layer that forms, it is conceivable to co-culture gut microbiota, pathogenic bacteria, or viruses with the intestinal stem cells to understand their impact on and dynamics within the mucus layer.

## METHODS

### Air–liquid interface culture of human intestinal stem cells

Human primary intestinal stem cells (generous donation from the Kuo Lab at Stanford, “XLS03”) were obtained using procedures previously described.^36^ Prior to seeding the cells on the Transwell inserts, the cells were maintained in an undifferentiated state by encapsulation in 40 *μ*l domes of EHS matrix, specifically Cultrex Basement Membrane Extract-Reduced Growth Factor (BME-RGF) Type 2 (R&D Systems, Minneapolis, MN), in 24-well plates. The 12 mm polycarbonate Transwell inserts with 0.4 μm pore size (Sigma-Aldrich, St. Louis, MO) were coated by adding EHS matrix diluted 50× in phosphate buffered saline (PBS) within the inserts and incubating for 1h at 37 °C. Using 5 mM ethylenediamine tetraacetic acid (EDTA) in PBS to dissolve the Culturex gels, the intestinal stem cells were then seeded onto Transwell inserts coated in EHS matrix at a seeding density of 60 000 cells/Transwell. The Transwells were then placed in 24-well plates, where 200 *μ*l and 500 *μ*l of IntestiCult^TM^ Organoid Differentiation Medium (Stemcell Technologies, Vancouver, Canada) was added below and inside the Transwell, respectively. The media was changed every other day until cells were confluent on the Transwell membrane. Then, only 500 *μ*l of media below the Transwell was changed every other day.

### IL-13 treatment of differentiated intestinal cells

For cultures receiving IL-13 treatment, the cultures were first cultured for at least 3 weeks until a robust mucus layer formed. Then, the cells were treated with 10 ng/ml IL-13 (Peprotech, Waltham, MA) for one week.^51^

### Immunocytochemistry

Cells were fixed and stained within the Transwell. To prepare samples for fixation, 200 *μ*l of PBS was used to collect any fluid inside the Transwell. The collected fluid was set aside for other assays. Cells were fixed by adding 150 *μ*l of 4% paraformaldehyde in PBS and incubating at RT for 1 h. Fixation solution was then aspirated and washed with PBS (3 × 5 min, RT). Cells were permeabilized for 30 min at RT with 0.2% Triton X-100 in PBS (PBST), washed with PBS (3 × 5 min, RT), then blocked for 3 h in PBS with 2% goat serum, 1% bovine serum album, 0.1% Triton X-100, 0.05% TWEEN-20, and 0.05% sodium azide. Primary antibody incubation was performed overnight at 4 °C. Antibody solutions were removed and washed in PBS (3 × 5 min, RT). Secondary antibody incubation was performed overnight at 4 °C. Secondary antibody solution was then removed and washed (3 × 5 min, RT). 1:2000 dilution of DAPI and 1:200 dilution of phalloidin were prepared in PBS and incubated for 1.5 h at RT, followed by a 5 min wash with PBS. The membrane was then cut from the Transwell insert by sliding a scalpel around the outer edge, dried of excess liquid, and placed with the cell-side down onto a droplet of ProLong Gold Antifade mounting medium on top of a rectangular coverglass. Mountant was allowed to cure for 18 h in the dark at RT before imaging on a TCS SPE confocal microscope (Leica, Wetzlar, Germany). Antibodies and stains used for this work are listed in Table S1 of the supplementary material.

### Quantitative real-time RT-PCR analysis

After washing the inside of the Transwell with 200 *μ*l of PBS, the membrane was cut from the Transwell insert using a scalpel and placed in a 1.5 ml Eppendorf tube containing 500 *μ*l of TRIzol reagent (Invitrogen, Carlsbad, CA) on ice to extract RNA. The solution was then vortexed to detach cells from membrane for optimal RNA extraction. Phenol-chloroform extraction was used to isolate RNA with Phase Lock Gels (Quantabio, Beverly, MA). A constant amount of RNA (0.1–1 *μ*g) measured via NanoDrop (Thermo Fisher Scientific, Waltham, MA) was reverse transcribed using the high-capacity cDNA reverse transcription Kit (Applied Biosystems, Foster City, CA). For qPCR, 6.6 *μ*l of cDNA in nuclease-free water was then mixed with 0.9 *μ*l of a 5 *μ*M forward and reverse primer pair (Integrated DNA Technologies, Coralville, IA) and 7.5 *μ*l of Fast SYBR Green Master Mix (Applied Biosystems, Foster City, CA) and run on StepOnePlus Real Time PCR System (Applied Biosystems). CT values were calculated using the StepOnePlus software (v.2.3) and analyzed by the ΔCT method. Primers used for this work are listed in Table S2 of the supplementary material.

### Dynamic light scattering microrheology

The Transwell was washed with 200 *μ*l of PBS before all fluid inside the Transwell insert was collected into a 1.5 ml Eppendorf tube. The collected fluid was then centrifuged at 9000 g for 3 min to collect the heavy phase at the bottom of the tube. The top 200 μl of fluid was removed. The remaining sample was mixed at a ratio of 1:9 of PEGylated polystyrene microspheres (500 nm diameter, Polysciences, Warrington, PA) to sample before being measured using dynamic light scattering (Malvern Panalytical, Malvern, UK) as previously described.^49^ Analysis of the scattering data were done using previously published and publicly available custom software (https://dlsur.readthedocs.io/).

### Magnetic microrheometry

Creep compliance experiments were conducted with a magnetic microwire rheometer as previously described.^42,43^ Briefly, a magnetic microwire probe of diameter 24 *μ*m and length 5 mm was deposited at the mucus–air interface of the culture. The Transwell was then placed between two electromagnetic coils in anti-Helmholtz configuration and mounted on an inverted microscope (Leica, Wetzlar, Germany) to permit live cell imaging and video of the probe motion during the experiment. A magnetic force was applied along the axis of the microwire by supplying current to the electromagnetic coils. The force was applied in a step function where the force was turned on for 10 s and off for 20 s twice over the course of 1 min. The ratio of the translational displacement of the microwire to the applied force was used to calculate the compliance vs time curves. A geometric resistance factor was used to account for the shear stress on the sample from the application of the magnetic force on the microwire probe. For hydrodynamic purpose, the microwire was approximated as a cylinder, and the cell layer was treated as a no-slip surface. The geometric resistance factor for a cylinder translating along the surface of a material with its axial axis parallel to a no-slip surface is given by $\frac{\pi L}{\ln[\frac{h}{r}+{\left[{\left(\frac{h}{r}\right)}^{2}-1\right]}^{1/2}]\;}$,^52,53^ where r and L are the radius and length of the microwire, respectively, and h is the height between the microwire axis and the no-slip surface. The height between the microwire and the cell surface, or the thickness of the mucus layer, was approximated from a z-stack of images of the sample, where images were taken from the bottom of the Transwell to the top of the microwire. The compliance data were then fit with the viscoelastic Burgers model to determine the steady state compliance and zero-shear viscosity. A stress sweep of applied forces from 95 to 220 nN was conducted. Across this range of applied forces, a consistent creep compliance was measured, leading to the conclusion that the measurements were in the limit of linear viscoelasticity (supplementary material, Fig. 4). All live cell rheology measurements shown in this work were made at an applied force of ∼180 nN.

### Statistical analysis

Statistical analyses for this study were performed using GraphPad Prism v.9.3.1 software. Pairwise t-tests were performed unless otherwise noted. For all studies, no marking or ns = not significant (p > 0.05), ^*^p < 0.05, ^**^p < 0.01, ^***^p < 0.001, and ^****^p < 0.0001.

## SUPPLEMENTARY MATERIAL

See the supplementary material for representative movies and figures of immunofluorescent staining of intestinal cell monolayers, zero-shear viscosity data, a representative video of microwire displacement on the mucus layer of an intestinal cell monolayer, and tables of the antibodies and primers used in this work.

## Data Availability

The data that support the findings of this study are available from the corresponding authors upon reasonable request.
